# Recent Advances in Intervention in Markovian Regulatory Networks

**DOI:** 10.2174/138920209789208246

**Published:** 2009-11

**Authors:** Babak Faryabi, Golnaz Vahedi, Aniruddha Datta, Jean-Francois Chamberland, Edward R Dougherty

**Affiliations:** 1 Department of Electrical and Computer Engineering, Texas A&M University, College Station, TX 77843, USA; 2 Department of Electrical and Computer Engineering, Texas A&M University, College Station, TX 77843, USA and Computational Biology Division, Translational Genomics Research Institute, Phoenix, AZ 85004, USA

**Keywords:** Regulatory networks, markovian decision processes, translational genomics, systems biology.

## Abstract

Markovian regulatory networks constitute a class of discrete state-space models used to study gene regulatory dynamics and discover methods that beneficially alter those dynamics. Thereby, this class of models provides a framework to discover effective drug targets and design potent therapeutic strategies. The salient translational goal is to design therapeutic strategies that desirably modify network dynamics *via* external signals that vary the expressions of a control gene. The objective of an intervention strategy is to reduce the likelihood of the pathological cellular function related to a disease. The task of finding an effective intervention strategy can be formulated as a sequential decision making problem for a pre-defined cost of intervention and a cost-per-stage function that discriminates the gene-activity profiles. An effective intervention strategy prescribes the actions associated with an external signal that result in the minimum expected cost. This strategy in turn can be used as a treatment that reduces the long-run likelihood of gene expressions favorable to the disease. In this tutorial, we briefly summarize the first method proposed to design such therapeutic interventions, and then move on to some of the recent refinements that have been proposed. Each of these recent intervention methods is motivated by practical or analytical considerations. The presentation of the key ideas is facilitated with the help of two case studies.

## 1. INTRODUCTION AND MOTIVATION

In biology, there are numerous examples where the (in) activation of one gene or protein can lead to a certain cellular functional state or phenotype. For instance, in a stable cancer cell line, the reproductive cell cycle is repeated and cancerous cells proliferate with time in the absence of intervention. One can use the *p53* gene if the intervention goal is to push the cells into apoptosis, or programmed cell death, or to arrest the cell cycle. The *p53* gene is one of the most well-known tumor suppressor genes, encoding a protein that regulates the expression of several genes such as *Bax* and *Fas/Apo1*, which function to promote apoptosis [1,2]. In cultured cells, extensive experimental results indicate that when *p53* is activated, e.g. in response to radiation, it leads to cell growth inhibition or cell death [3]. The *p53* gene is also used in gene therapy, where the target gene (*p53* in this case) is cloned into a viral vector. The modified virus serves as a vehicle to transport the *p53* gene into tumor cells to generate intervention [4,5]. As this and many other examples suggest, it is prudent to use regulatory models to design therapeutic interventions that expediently modify the cell's dynamics *via *external signals. These system-based intervention methods can be useful in identifying potential drug targets and discovering treatments to disrupt or mitigate the aberrant gene functions contributing to the pathology of a disease.

In [6,7], several methods to design therapeutic interventions are discussed. Some of these methods are intended to reduce the likelihood of the gene-expression profiles associated with aberrant cellular functions *via *manipulation of a control gene. In a nutshell, whenever changing the expression level of a control genes, e.g. *p53* in the above scenario, is perceived as a therapeutic option, these system-based therapies search for the most effective sequence of such changes to beneficially alter cell dynamics. The resulting intervention strategy specifies the appropriate expression of the control gene in order to reduce the likelihood of pathological cellular functions.

In the case of a cancerous tumor, the objective of treatment could be to diminish the long-run likelihood of metastasis. In this scenario, one may consider the correlation between metastasis and the abundances of messenger RNA for certain genes. For instance, the abundance of messenger RNA for the gene *Wnt5a* has been found to be highly discriminating between cells with properties typically associated with high versus low metastatic competence [8]. In this case, the messenger RNA level of the gene *Wnt5a* can be used to annotate profiles of gene expressions as desirable and undesirable. One partition of gene-expression profiles corresponds to high, while the other to low, metastatic competence. Having defined a cost function to discriminate between the two sets of gene-expression profiles, the task  of finding an effective intervention strategy can be mathematically formulated as a sequential decision making problem for a pre-defined cost of intervention. The objective of the decision maker is to identify a strategy that minimizes a well-defined function of the accumulated cost over time. Such a strategy can be seen as a system-based treatment to avoid undesirable gene-expression profiles contributing to the pathology of a disease.

The aforementioned method of devising therapeutic interventions has been studied in the context of probabilistic Boolean networks [9]. Probabilistic Boolean networks are a class of discrete-time discrete-space Markovian regulatory networks in which all the genes in the model are assumed to be updated simultaneously. Major efforts have been focused on devising intervention strategies that affect the dynamics of probabilistic Boolean networks. The effect of an intervention strategy that is beneficial in the short-term may wear out over time. Thus, it is important to look for intervention strategies that consider the long-run effects. In the framework of probabilistic Boolean networks, the theory of infinite-horizon stochastic control has been employed to find optimal intervention strategies with respect to the defined objective functions [6]. An optimal strategy determines the actions to be taken using the external signal in response to each gene-expression profile.

Formulating the problem of intervention in a regulatory network as a classical infinite-horizon decision making process, which we refer to as *classical intervention* throughout this article, introduces an elegant analytical framework that may be instrumental to enhance our understanding of treatment discovery. Despite its conceptual benefits, the classical intervention fails to address many practical and technical issues. In the past few years, the classical framework has been extended in several directions to improve system-based intervention schemes. To achieve this goal, we have envisaged a number of objectives for which both methodology and techniques must be improved. These proposed analytical tools provide insight into the design of effective therapeutic interventions. These methods strive to address some of the practical concerns that are brought up by medical practitioners. The aim of this article is to highlight the objectives behind each proposed method and briefly explain it *via *a biological case study. This article also aims to motivate further research in this area. We are aware that, despite progress in this area, extensive research is required before decision making processes can be fully integrated into medical practice. Before proceeding further, we briefly discuss the motivation behind each of the schemes discussed in this paper.

### 1.1. Model-free Intervention Design

Sequential decision making techniques can be categorized into two classes. The first class of schemes, such as the classical intervention, requires exact optimization of a cost function by the decision maker to find an effective treatment within the space of all possible treatments. It is well-known that the exact solution of such a search problem is not robust relative to inaccuracy of the underlying network model. The procedures for regulatory network inference are prone to modeling errors. They suffer from insufficient empirical measurements and computational complexity [10]. In addition, the computational complexity of sequential decision making optimization prohibits its use with regulatory models possessing large numbers of components. To bypass the impediment of model inference and to mitigate the numerical problems associated with the exact optimization approach, heuristic schemes can be used to design system-based therapies. A heuristic intervention method estimates insightful statistics of the regulatory network from empirical measurements and utilizes these statistics to greedily search the space of all strategies for an effective one. Typically, effective heuristic methods provide lower computational complexity, are robust to modeling errors, and also adapt to changes in the underlying biological system.

In this manuscript, we explain two heuristic intervention schemes. First, we consider an intervention strategy based on reinforcement learning [11]. Given the cost structure, the reinforcement intervention estimates the statistics of the cost function and uses this information to learn an effective strategy. The second heuristic approach is based on mean first-passage time statistics in Markovian processes. This scheme utilizes the estimated mean first-passage times to devise a greedy strategy based on a simple consideration: postponing transition to gene-expression profiles associated with aberrant cellular functions as long as possible. Both heuristic strategies progressively improve their performances as more empirical measurements become available.

### 1.2. Limiting the Side-effects of Therapies

Besides confronting inferential, complexity, and robustness problems, which are essentially engineering issues, one also needs to take into account practical medical issues. Consider the fact that medicine is able to exploit the biochemical differences between bacteria and human cells so as to achieve toxic drug concentrations in the former while sparing the latter. This selectivity largely contributes to the success in treating bacterial infections. Unfortunately, such high selectivity is at present elusive in the treatment of human cancers. Hence, great efforts are required to determine dose schedules that maximize the benefit to toxicity ratio in cancer therapy [12]. Dose intensity is a measure of treatment delivery that looks at the amount of drug delivered per unit of time. To mitigate the detrimental side effects of a treatment in general, we should account for dose intensity in a system-based intervention method. Therapeutic intervention should avoid undesirable gene-expression profiles while accounting for the quantity or frequency of applied drugs. A higher drug dose intensity can be delivered by increasing the dose per cycle (dose escalation) or by reducing the interval between cycles (dose density).

Cancer treatments are generally given in *cycles*: each treatment is followed by a *recovery phase*. Tumors, given less time to grow between treatments, are more likely to be eradicated. Administering high quantities of drugs at the beginning of a chemotherapy cycle might fail for two reasons. First, levels higher than a certain concentration may not increase the killing rate of cancer cells. Second, even if they did, the toxicity could be intolerable to the patient. In practice, optimizing the schedule means determining a way to give the maximum integrated effect over as short a time as possible, consistent with a reasonable quality of life [12]. To this end, we consider a *cyclic intervention* method to amend the classical intervention method and address this practical concern. Cyclic intervention can be utilized to design effective therapeutic strategies when each treatment is permitted only after a recovery phase.

Dose intensity can also be regulated by the number of interventions in a therapeutic strategy. A treatment based on estrogen is often used by women after menopause to alter their accelerated aging trend. The amount of estrogen received during this treatment should not exceed a threshold. An overdose may increase the chance of developing breast and ovarian cancers. While this phenomenon is not fully understood, it is conceivable that estrogen therapy may have side effects on gene regulation. Estrogen generates two types of complexes through binding to two classes of receptors. The generated complexes are transported into the nucleus to bind to the enhancer elements on the target genes with the help of a coactivator. The coactivator is also required for efficient transcriptional regulation by estrogen. This function, in cooperation with a coactivator acts like a transcription factor, affecting target genes such as the *PENK* gene [13]. Two types of receptors are competing for binding to the estrogen received *via* treatment [14]. The first type of complex binds DNA better but performs less efficiently to bind the coactivator; the second type of complex binds the coactivator better but performs poorly when binding DNA. When the level of estrogen is below a threshold, there is no competition for DNA binding. Hence, the second type of complex also binds DNA and activates the downstream target gene *PENK*, with the help of the coactivator. However, when the estrogen level is high, both types of complexes exist at high concentrations and the second type of complex binds and depletes the coactivator. The level of coactivator available to complex type one drops. Hence, the complex type one does not have necessary coactivator, and has a small chance to bind to DNA and cause activation of gene *PENK*. If the *PENK* gene plays a role in tumor suppression, for instance, then this could explain why high levels of estrogen have a tumorigenic effect. An appropriate treatment strategy mitigates this problem by bounding the expected number of treatments received by a patient and, as a result, limits the dose intensity of estrogen.

In general, the likelihood of eradicating pathological  cell functions is maximized by delivering the most effective dose intensity of a drug whose toxicity can be tolerated  by the patient. The dose intensity of a drug is directly related to the number of interventions in a therapeutic strategy. *Constrained intervention* is introduced to incorporate the aforementioned concern in the system-based therapy design paradigm. Using constrained intervention methods, we seek an effective regulatory treatment that reduces the likelihood of visiting undesirable gene-expression profiles in the long run while providing an upper bound on the expected number of interventions a patient can receive [15].

### 1.3. Accommodating Different Biological Time Scales

The scheme to update the gene values in a regulatory model plays a crucial role in its ability to describe the dynamics of gene interactions and thereby influences the effectiveness of designed therapies. Common to the previously cited intervention methods is the assumption that the genes are updated synchronously in the underlying network. From a biological perspective, interactions among genes and proteins causing various processes occur over a wide range of time-scales. In a synchronous model, the tacit assumption is that asynchronous updating will not unduly alter the presented biological properties central to the application of interest. This assumption may not generally hold. Various potential issues with synchronous networks have been raised [16-18]. 

These observations motivate us to study intervention based on discrete state-space models that can capture timing information in gene interactions. An asynchronous Markovian regulatory model, suited to our intervention objective, should posses four characteristics: (1) it should be inferable from the empirical time-course measurements; (2) it should accurately represent relations among genes of interest; (3) it should enable us to analytically study the temporal behavior of relevant phenomena; and (4) the model should be appropriate for the study of therapeutic intervention. With these conditions in mind, we have proposed two asynchronous Markovian regulatory networks [16]. In the first asynchronous framework, the updating period of each gene is fixed, but can differ from one gene to another. The second asynchronous regulatory model introduces asynchronism relative to the state-space of gene-expression profiles. This approach is more suitable from the inference perspective. In Section 6, we will briefly describe these models and determine how they can be used as a tool to search for effective therapeutic interventions. These asynchronous models can potentially provide more effective intervention strategies, depending on our ability to perform satisfactory inference. To date, the lack of sufficient time-course data has prohibited the inference of any realistic asynchronous networks; however, the situation is expected to improve in the future with the advent of new experimental techniques.

## 2. A MARKOVIAN REGULATORY MODEL

Constructing complex regulatory models that finely describe the interactions among biological components related to a disease requires precise understanding of the underlying biological processes and extensive amounts of experimental data. Although genetically pathological cells, such as cancer cells, have been extensively studied, we  still lack sufficient knowledge and data sets to construct  such complex models. Meanwhile, it is equally important, especially from a translational perspective, to discover effective drug targets and devise therapeutic strategies with the help of simpler regulatory models, such as Markovian regulatory models. To this end, probabilistic Boolean networks, which compose a class of discrete-time discrete-space Markovian regulatory networks have been utilized  to devise system-based therapeutic interventions. This class of rule-based models, which allow the incorporation of uncertainty into inter-gene relationships, are probabilistic generalizations of classical Boolean networks [19,20].

In a rule-based regulatory network, such as a Boolean network, a *regulatory graph* describes the multivariate interactions among the components. In a genetic regulatory network, the vertices of a regulatory graph are the genes. A directed edge starts from a predictor gene and ends at an influenced gene. All the genes directed to a node are its predictors. A *regulatory rule* defines the multivariate effects of the predictors on the gene. The gene values are selected from a set of possible quantization levels to facilitate the modeling of gene interactions by logical rules. Strong evidence suggests that discrete-state-space models are capable of describing interactions between biological components [21,22].

If genes values are quantized to two levels, then the rule-based networks are described by a collection of Boolean functions, with 0 or 1 meaning genes are OFF or ON, respectively. Ternary quantization arises when we consider individual genes to be down-regulated, up-regulated, or invariant. This situation commonly occurs with cDNA microarrays, where a ratio is taken between the expression values on the test channel (red) and the base channel (green). In this paper, we will develop the methodology for the binary case, so that gene values are either 0 or 1. The methodologies are nevertheless applicable to any finite quantization level. Fig. (**1**) shows the regulatory graph of a hypothetical three-gene network. There is a unidirectional relation between genes *x_1_* and *x_3_*. The relation between genes *x_1_* and *x_2_* is bidirectional.

To completely specify a class of regulatory networks, we need to adopt an updating scheme. Once this is accomplished, we can translate the dynamical information of the regulatory graph and the regulatory rules into an *oriented graph*. The vertex of an oriented graph is a *state*, which  is the aggregated values of all the genes at a given time.  An edge traverses from one state to another state of  an oriented graph if a transition can occur in the direction  of the edge from one vertex to the other. The choice of  the updating scheme plays a crucial role in the dynamical behavior of the network. In Boolean networks, the values  of genes are updated synchronously at equally distant updating epochs. For instance, Figs. (**1**) and (**2**) show a pair of oriented and regulatory graphs. According to this oriented graph, whenever the aggregate value of the three genes in the network is ( *x*_1_ = 0 , *x*_2_ = 0 , *x*_3_ = 0 ) and if all the genes are updated synchronously, then the next state is ( *x*_1_ = 0 , *x*_2_ = 0 , *x*_3_ = 1).  A regulatory graph is a static representation of interactions among biological components, whereas an oriented graph shows the dynamics of the interactions among these components. A key point concerning Boolean networks is that, in the long run, the network will settle into an *attractor cycle* (e.g. “001” in Fig. (**2**)), meaning that the network will endlessly cycle through some set of states.

To incorporate the effect of latent variables outside the model, whose behaviors influence regulation within the system, stochasticity is introduced into the model by allowing several possible regulatory functions for each gene and allowing random modification of the genes. The resulting model is called a *probabilistic Boolean network* (PBN) [9], where the terminology *Boolean* refers to the logical character of the relations, not that they are necessarily binary. If the regulatory functions are allowed to change at every time point, then the PBN is said to be *instantaneously random* [9]. On the other hand, in a *context-sensitive* PBN, function updating only occurs at time points selected by  a binary random switching process [23,24]. In essence, a PBN is composed of a collection of networks (oriented graphs); between switches it acts like one of the constituent networks (oriented graphs), each being referred to as a *context*. The switching frequency of the context differentiates the instantaneously random PBN from the context-sensitive PBN. The PBN model also allows random perturbation of genes at each updating instant. By definition, the attractors of a PBN consist of the attractors of its constituent contexts.

The oriented graph of a PBN is a Markov chain [9,25]. The transition probabilities associated with a PBN act on its states and describe their trajectories over time. For an instantaneously random PBN, the state consists of a *gene-activity profile* (GAP), which presents the aggregated expressions of all the genes in the PBN at each instant; for a context-sensitive PBN, the state includes a GAP and a context. A PBN with *n* genes has 2^*n*^ GAPs. We denote the set of all possible GAPs by *χ*. By the Markovian property, the dynamic behavior of an instantaneously random PBN whose states are in *χ* can be represented as a discrete-time equation

(1) $$x\left(t+1\right)=f\left(x\left(t\right),w\left(t\right)\right)t=0,1,...$$

where $x\left(t\right)$ is the state at the updating epoch *t*. The disturbance $w\left(t\right)$ is the manifestation of uncertainties in the biological system, due either to context switching or change in the genes resulting from random gene perturbation.

Similarly, the oriented graph of a context-sensitive PBN can be described by a discrete-time equation

(2) $$z\left(t+1\right)=f\left(z\left(t\right),w\left(t\right)\right)t=0,1,...$$

where the state $z\left(t\right)$ is an ordered pair consisting of a constituent network *κ* and a GAP *x*. The set $z=\left\{\left(\kappa ,x\right):\kappa \in \left\{1,...,k\right\},x\in \chi \right\}$ denotes the state-space of the oriented graph associated with a context-sensitive PBN [25]. The number of states in *z* is $2^{n}\times k$ for a context-sensitive PBN with *n* genes and *k* contexts. The disturbance $w\left(t\right)$ is the manifestation of uncertainties in the model determined by the possibility of switching the contexts, the probability distribution of selecting contexts after a switching event, and the probability of changes in gene status resulting from random perturbation.

In the remainder of this section, we present two case studies which are used throughout the paper. We construct a PBN for each case. The first PBN is constructed using gene-expression data collected in a profiling study of metastatic melanoma [11]. The second PBN is obtained by suitably extending a Boolean model proposed in [26] for modeling mammalian cell cycle regulation. The second network postulates the mammalian cell cycle with a mutated phenotype [16].

### 2.1. Metastatic Melanoma Gene Expression

In this sub-section, we construct an instantaneously random PBN based on steady-state data collected in a profiling study of metastatic melanoma in which the abundance of messenger RNA for the gene *Wnt5a* was found to be highly discriminating between cells with properties typically associated with high metastatic competence versus those with low metastatic competence [8]. These findings were validated and expanded in a second study, in which experimentally increasing the levels of the *Wnt5a* protein secreted by a melanoma cell line *via *genetic engineering methods directly altered the metastatic competence of that cell as measured by the standard *in vitro* assays for metastasis [27]. A further finding of interest in this study is that an intervention that blocks the *Wnt5a* protein from activating its receptor, with the help of an antibody that binds the *Wnt5a* protein, can substantially reduce *Wnt5a*'s ability to induce a metastatic phenotype. This suggests intervention based on a strategy that alters the contribution of the *Wnt5a* gene to biological regulation. Disruption of this influence can potentially reduce the chance of a melanoma metastasizing, a desirable outcome. Ten genes, including the *Wnt5a* gene, were selected in [28] based on the predictive relationships among 587 genes: *Wnt5a*, *pirin*, *S100p*, *Ret1*, *Mmp3*, *Phoc*, *Mart1*, *Hadhb*, *Synuclein*, and *Stc3*. We apply the design procedure proposed in [29] to generate an instantaneously random PBN possessing four constituent BNs. The method of [29] generates BNs with given attractor structures and the overall PBN is designed so that the data points, which are assumed to come from the steady-state distribution of the network, are attractors in the designed PBN. The regulatory graphs of these BNs are given in  Fig. (**3**). In the binary representation of the GAPs, the order of the genes is as listed earlier with *Wnt5a* being the most significant bit and *Stc3* being the least significant bit.

The intervention objective for this 10-gene network is to down-regulate the *Wnt5a*, because this gene ceasing to be down-regulated is strongly predictive of the onset of metastasis. We will present two intervention methods using the constructed PBN, with the aim of down-regulating the *Wnt5a* gene. This model is used because the relation of *Wnt5a* to metastasis is well established and the binary nature of the up or down regulation suits a binary model. The first step in devising any therapeutic intervention is to designate desirable and undesirable states. This depends upon the existence of relevant biological knowledge. In this particular example, the use of the status of *Wnt5a* has resulted from prior biological knowledge relating the status of this gene to metastasis in melanoma tumors. A GAP is desirable if *Wnt5a* is 0 and undesirable if *Wnt5a* is equal to 1.

### 2.2. Mutated Mammalian Cell Cycle

Next, we consider a context-sensitive PBN that is a probabilistic generalization of the Boolean model proposed in [26] for mammalian cell-cycle regulation. This context-sensitive PBN postulates the mammalian cell cycle with a mutated phenotype. Mutated cells grow in the absence of extra-cellular growth factors [16].

During the late 1970s and early 1980s, yeast geneticists identified the cell-cycle genes encoding for new classes of molecules, including the cyclins (so-called because of their cyclic pattern of activation) and their cyclin dependent kinase (cdk) partners [26]. Our model is rooted in the work of Faure *et al.*, who have recently derived and analyzed the Boolean functions of the mammalian cell cycle [26]. Using these Boolean functions, the authors have been able to quantitatively reproduce the main known features of the wild-type biological system, as well as the consequences of several types of mutations.

Mammalian cell division is tightly controlled. In a growing mammal, the cell division should coordinate with the overall growth of the organism. This coordination is controlled *via *extra-cellular signals. These signals indicate whether a cell should divide or remain in a resting state. The positive signals, or growth factors, instigate the activation of Cyclin D (*CycD*) in the cell.

The key genes in this model are *CycD*, retinoblastoma (*Rb*), and *p27*. *Rb* is a tumor-suppressor gene. This gene is expressed in the absence of the cyclins, which inhibit *Rb* by phosphorylation. Whenever *p27* is present, *Rb* can be expressed even in the presence of *CycE* or *CycA*. Gene *p27* is active in the absence of the cyclins. Whenever *p27* is present, it blocks the action of *CycE* or *CycA*. Hence, it arrests the cell cycle. Table **1** summarizes the Boolean functions of the wild-type cell cycle network.

The preceding explanation represents the wild-type cell-cycle model. Following one of the proposed mutations in [26], we assume that *p27* is mutated and its logical rule is always zero (OFF). In this cancerous scenario, *p27* can never be activated. As we mentioned earlier, whenever *p27* is present, *Rb* can be expressed even in the presence of *CycE* or *CycA*. For the mutated cell-cycle network, however, *p27* is always zero and *Rb* cannot be expressed in the case where *CycD* is not present but *CycE* or *CycA* is active. This mutation introduces a situation where both *CycD* and *Rb* might be inactive. As a result, in this mutated phenotype,  the cell cycle continues in the absence of any growth factor. In other words, we consider the states in which both *Rb*  and *CycD* are down-regulated as undesirable states, when *p27* is mutated. Table **2** summarizes the mutated Boolean functions.

The Boolean functions in Table **2** are used to construct a context-sensitive PBN model for the cell cycle [16]. The extra-cellular signal to the cell-cycle model is considered to be a latent variable. The growth factor is not part of the cell and its value is determined by the surrounding cells. The expression of *CycD* changes independently of the cell's content and reflects the state of the growth factor. Depending on the expression status of *CycD*, we obtain two constituent Boolean networks for the PBN. The first constituent Boolean network is determined from Table **2** when the value of *CycD* is equal to zero. Similarly, the second constituent Boolean network is determined by setting the value of *CycD* to one. To completely define the context-sensitive PBN, the probability of switching the context, the probability that a gene perturbs, and the probability distribution of selecting each constituent network have to be specified. We assume that these are known. Here, we set the switching and the perturbation probabilities each equal to 0.001, and assume that the two constituent networks are equally likely.

According to Table **2**, the mutated cell-cycle PBN consists of nine genes: *CycD*, *Rb*, *E2F*, *CycE*, *CycA*, *Cdc20*, *Cdh1*, UbcH10, and *CycB*. The above order of genes is used in the binary representation of the states of the context-sensitive PBN, with the context of the networks, *CycD*, as the most significant bit. *Rb* is in the most significant position in the gene-activity profiles and *CycB* is placed at the least significant bit.

Choosing *CycD* and *Rb* as the most significant bits in the state representation of the context-sensitive PBN facilitates the characterization of the undesirable states. We assume that the simultaneous down-regulation of *CycD* and *Rb*, i.e. the cell growth in the absence of growth factors, is undesirable. Consequently, the state-space is readily partitioned into undesirable and desirable states. As mentioned earlier, application of any system-based therapeutic method with external control requires the designation of desirable and undesirable states, and this depends upon the existence of relevant biological knowledge. In the cell-cycle example when *p27* is mutated, the functionality of the network suggests that the states in which both *Rb* and *CycD* are down-regulated should be avoided.

## 3. CLASSICAL THERAPEUTIC INTERVENTION

In PBNs, where genes are updated simultaneously, appropriate transition probability matrices that act on the states of the oriented graphs are sufficient to fully describe the dynamics in equations (1) and (2). Methods have  been proposed that use information in these probability distributions to devise effective therapeutic strategies [6,7]. Among these, an infinite-horizon sequential decision making method has been proposed to design therapeutic strategies. Altering the long-run likelihood of states favorable to a pathological cell functionality is the objective of the decision maker. To this end, the task of finding an effective intervention strategy has been formulated as a classical sequential decision making optimization. For a pre-defined cost of intervention and a cost-per-stage function that discriminates between the states of the system, the objective of the decision maker is to minimize the accumulated expected cost associated with the progression of the system. That is, given the state of the system, an effective intervention strategy identifies which action to take so as to minimize the overall expected cost. Consequently, the devised intervention strategy can be used as a therapeutic strategy that alters the dynamics of aberrant cells to reduce the long-run likelihood of undesirable states favorable to the disease.

To be more precise, in the presence of an external regulator, it is assumed that the PBN has a binary intervention input $u\left(t\right)$ at each epoch *t*. The intervention input $u\left(t\right)$ , which takes values in set $C=\left\{0,1\right\}$ , specifies the action on a control gene. Treatment alters the status of the control gene, which can be selected among the genes in the network. If treatment is applied, $u\left(t\right)=1$ then the state of the control gene is toggled; otherwise the state of the control gene remains unchanged. To completely define the decision making procedure, a cost-per-stage is associated to each possible event. In general, the cost-per-stage at each instant *t* depends on the current state and the action of an external signal at that instant. Given the system is initiated from state ***z***_0_, the sequential decision maker searches for an optimal strategy $\pi^{\text{*}}$ , one that minimizes the expected cost aggregated over the long-run progression of the PBN. In other words, in an infinite-horizon intervention problem, we seek an admissible intervention strategy $\pi^{\text{*}}$ that minimizes the expected total cost for each initial state ***z***_0_:

(3) $$\pi^{*}\left(z_{0}\right)=\text{arg}\underset{\pi }{\text{ min }}J_{\pi }\left(z_{0}\right)$$

where $J_{\pi }\left(z_{0}\right)$ is the expected total cost aggregated over the long run. A strategy *π* is a sequence of decision rules for each updating epoch *t* acting on a control gene. In general, a decision rule at updating epoch *t* selects action $u\left(t\right)$ according to the history of the system as well as the current state.

## 4. MODEL-FREE INTERVENTIONS FOR META-STATIC MELANOMA

The classical intervention described in Section 3 requires exact optimization of the cost function (3). The effectiveness of a strategy devised from the solution of (3) depends on  the accuracy of the underlying regulatory network. More-over, the computational complexity of optimization problem (3) increases exponentially with the number of genes in  the model. To mitigate this numerical challenge and bypass the impediment of model estimation, two heuristic methods have been proposed. One is based on reinforcement learning (RL) [11], and the other is based on mean first-passage  times (MFPT) [30]. In the framework of Markovian regulatory networks, these heuristic intervention schemes learn effective strategies from two different statistics of the model.

Given the cost structure, RL intervention learns an effective strategy based on several generated trajectories of states and applied actions. These empirical measurements are used to estimate the average aggregated cost ***J**π*(z_0_) with respect to various actions and observed states. The RL scheme yields an effective therapeutic strategy, while possessing constant complexity with respect to the number of genes [11]. It has been shown that in the scenario when  a large number of measurements is available the RL strategy converges to the strategy devised by the classical intervention.

The MFPT intervention devises an effective strategy based on two greedy actions: (1) it is preferable to *reach* desirable states as quickly as possible; (2) it is preferable to *leave* undesirable states as early as possible. Given a control gene, if without intervention, a trajectory originating from a particular desirable state reaches the set of undesirable states on average faster than when there is a one-time intervention, then it is reasonable to intervene and force the trajectory of the model to initialize from the new state specified by the intervention. Similarly, if without intervetion, the trajectory originating from an undesirable state reaches the set of desirable states on average faster than when there is a one-time intervention, then it is reasonable not to intervene. These insights motivate the design of intervention strategies using mean first-passage times, since the latter are precisely the statistics used to quantify the average time to transition from a state to a set of states. When time-course measurements are available, the mean first-passage times from each desirable state to the set of undesirable states and, vice versa, from each undesirable state to the set of desirable states,  can be estimated. The MFPT algorithm then utilizes these estimated statistics to devise its heuristic strategy.

A salient feature in both heuristic methods [11] and [30] is that they are *model-free*, i.e. they do not require perfect knowledge of the model parameters. We should point out that it is still assumed that the dynamics of the underlying system are modeled as a Markovian network. The term *model-free* implies that it is not required to estimate the parameters of the PBN explicitly. As explained in Section 3, the intervention method for PBNs is model dependent, requiring at least the knowledge of the transition probability matrix associated with the underlying PBN. This can be derived from the PBN if the latter is known. Since in practice PBNs are not known except *via *system identification from experimental data, one is faced with a difficult inference problem [10]. This problem can be avoided by directly inferring the transition probability matrix; however, this is still a formidable task because the complexity of estimating the transition probabilities of a Markov chain increases exponentially with the number of genes in the model. When time-course measurements are available, the RL and MFPT strategies can be implemented directly from the empirical measurements. Hence, they have low complexity, are robust to modeling errors, and are also adaptive to changes in the underlying biological system.

The RL and MFPT strategies are employed to control the *Wnt5a*-related network described in Section 2.1. The performances of these two heuristic methods are also compared to that of the classical intervention. As noted in Section 2.1, down-regulation of *Wnt5a* is a reasonable objective for an intervention strategy. The RL and MFPT strategies are applied to the inferred PBN. Here, *pirin* is chosen as the control gene [11]. As performance measures, we consider the percentages of reduction in the total probability of the undesirable states in the long run when the classical, RL, and MFPT strategies are applied; these being denoted by Δ*P*^opt^, Δ*P*^RL^, Δ*P*^MFPT^, respectively. It should be emphasized that the strategy of the classical intervention is optimal with respect to the cost function, since the full model is assumed to be known to the decision maker.

Time-course measurements for 10^6^ time-steps from the PBN constructed for the melanoma case study are generated. The application of the RL and MFPT strategies are preceded by a learning duration of 10^*k*^ measurements, with *k* = 3,...,6. Hence, Δ*P*^RL^ and Δ*P*^MFPT^ are functions of the learning duration. On the other hand, Δ*P*^opt^ is computed from the PBN by directly solving the optimization problem (3). Fig. (**4**)  shows the differences between Δ*P*^RL^ and Δ*P*^MFPT^ with Δ*P*^opt^ as functions of the logarithm of the learning duration. After 10^3^ measurement points, the difference between the performance of MFPT and the classical strategy is 0.114 , while this difference is 0.166 in the case of the RL strategy. In particular, for a lower number of observations, which corresponds to a more realistic scenario, the MFPT intervention outperforms the RL intervention. On the other hand, after 10^6^ measurement points, the difference between the performances of the MFPT scheme and the classical method is 0.003, while the same difference is 0.002 in the case of the RL method. This shows that as the size of the training data increases, the RL strategy outperforms the MFPT intervention strategy. Hence, the amount of available data would be a deciding factor in choosing between these two heuristic schemes.

Existing technologies are not capable of providing sufficient empirical measurements for inference of PBN parameters explicitly [10]. To reduce the inference problem, one might directly estimate the Markov chain associated with the PBN, which is all that is required for the classical intervention method. The performance of the resulting intervention strategy will depend on the accuracy of the estimation. We have conducted another set of experiments  to compare the performance of the MFPT scheme with  an optimal strategy devised from the estimated transition probability matrix of the Markov chain. We generate synthetic time-course data for 100,000 time-steps from  an existing model. Using the synthetic time-course data,  we estimate the mean first-passage times after each 10*^k^* time-steps, for *k* = 2,...,5. As the duration of estimating the mean first-passage times increases, Δ*P*^MFPT^ approaches Δ*P*^opt^ Fig. (**5**) shows the average of Δ*P*^opt^ - Δ*P*^MFPT^, where Δ*P*^opt^ is obtained from the transition probability of the actual PBN, for various learning durations over 1000 trials. For an optimal policy based on the Markov chain estimated from the data, we denote the shift in the steady-state distribution by Δ*P*^opt*^. Fig. (**5**) shows the average of Δ*P*^opt^ - Δ*P*^opt*^ with various learning durations over 1000 trials. The graphs clearly demonstrate the superior performance of the modelfree approach using the MFPT algorithm. The MFPT scheme using 100 measurements outperforms the intervention strategy devised from an estimated Markov chain using 100, 000 observations.

## 5. LIMITED-SIDE-EFFECT INTERVENTIONS FOR MUTATED MAMMALIAN CELL CYCLE

In the classical intervention, at each epoch a devised strategy decides whether to intervene or not in order to reduce the likelihood of undesirable states without imposing any restrictions on the quantity or frequency of applied treatments. In medical practice, however, dose intensity in a treatment is limited to mitigate the detrimental side effects of therapy. Here, we describe two approaches that amend the unrestricted classical intervention strategy with the goal of accommodating such constraints. In the first approach, referred to as cyclic intervention, the frequency of applying treatments is adjustable with each treatment being followed by a recovery phase that allows the side effects to subside [31]. Using the mutated cell-cycle network as an example, we explain how to design an effective cyclic therapeutic strategy when the treatment is only permitted after a fixed recovery phase. An alternative approach to control side effects is to bound the quantity of the prescribed interventions [15]. To determine the best integrated effect consistent with a reasonable quality of life, we seek an effective therapeutic method that reduces the likelihood of states related to an undesirable cell functionality by minimizing the associated cost function, while providing an upper bound on the expected number of interventions received by a patient. Once again a constrained intervention strategy for the mutated cell-cycle network is designed.

### 5.1. Cyclic Therapeutic Intervention

Certain types of cancer therapies, such as chemotherapy, are given in cycles with each treatment being followed by a recovery period. During the recovery period, the side effects tend to gradually subside. In the classical intervention framework, at every state transition of the system, the intervention strategy dictates whether to apply treatment or not. The objective in [31] is to devise an effective intervention strategy under the constraint that intervention is permitted only every *W* transitions, where *W* denotes the length of the recovery period. A classical intervention strategy that is optimal for the case where intervention is permitted at every transition is not necessarily optimal (i.e. may not minimize the expected total cost) if one is only permitted to apply treatment every *W* transitions. We refer to the strategy that is optimal when intervention is permitted every *W* transitions as a *cyclic* strategy.

A *treatment window* is defined to be every *W* transitions of the system. Intervention is permitted at the beginning of a treatment window. Thereafter, the system transitions *W*–1 steps without intervention. To impose the cyclic constraint on strategies, a new Markov chain is constructed with an augmented state space based on the original Markov chain of the mutated cell-cycle network. A cyclic strategy can be found by solving the stochastic control problem for the Markov chain with the augmented state space *via *classical dynamic programming algorithms. However, this procedure may be prohibitive due to the size of the augmented state space. To address this issue, it has been shown that the augmented state space can be collapsed to a compressed state space of size equal to the original state space [31]. This reduction in the size of the state space is accomplished by properly accumulating the expected cost of the system progression during the recovery period. The new cost function is used to select the proper action at the time instants when intervention is permitted. In [31], the convergence of the corresponding dynamic programming algorithm is established, and it is also shown how the cyclic intervention strategy can be devised.

In [31], the cyclic intervention in the mutated mammalian cell-cycle network is demonstrated. The resulting reduction in the likelihood of undesirable states, *ΔP^W^*, and the *average* total cost for both the classical and the cyclic strategies are shown in Figs. (**6**) and (**7**), respectively. It is evident from these figures that in the long run less treatment is applied for a larger treatment window and consequently a higher cost is induced. Hence, the average costs of both the classical and cyclic strategies increase as the treatment window increases. Figs. (**6**) and (**7**) show that, for the mutated cell-cycle network, the classical strategy approximates the optimal cyclic strategy quite well. However, this may not hold in general. Indeed, our comprehensive numerical studies for various network structures in [31] shows that, in general, these two intervention strategies exhibit different properties.

### 5.2. Constrained Therapeutic Intervention

Cancer treatment may include the use of chemotherapy, radiation therapy, targeted gene therapy, etc. All of these treatment options are directed at killing or eradicating cancerous cells. Unfortunately, cancer treatments may also damage healthy cells. This results in complications and harmful side effects. It is therefore desirable to restrain the side effects of a treatment. This goal can be achieved by enforcing an upper bound on the expected number of treatments a patient may receive during therapy. A classical intervention strategy, devised by solving the unconstrained optimization problem (3), reduces the chances of visiting undesirable states; however, it does not provide a mechanism for constraining the frequency of applying treatments within the resulting intervention strategy. To address this shortcoming, constrained intervention is introduced by imposing an appropriate constraint on the optimization problem (3).

This is accomplished by associating another cost-perstage *c(z,u)* with each state-action pair (*z,u*). This new cost-per-stage should be defined to appropriately reflect the intended constraint. Specifically, we bound the expected number of interventions in the long run to limit the dose intensity of an intervention within a prescribed treatment. To this end, we define the total constraining cost given a strategy *π* and an initial state z_0_ as **C***_π_* (z_0_). Having this new constraining cost function, we reformulate the unconstrained intervention problem (3) in the mutated cellcycle model as a constrained intervention,

(4) $$\underset{\pi }{\text{min}}J_{\pi }\left(z_{0}\right),\text{ such that }C_{\pi }\left(z_{0}\right)\leq C_{\text{total}},$$

where ***C**_total_* is the upper bound on the expected number of interventions in the long run.

The complete treatment of the constrained optimization problem (4) is presented in [15]. In that reference, it is shown that the objective function ***J**_π_*(z_0_) and the constraining cost function ***C**_π_*(z_0_) can be presented as a linear combination of the *occupation measure* and the cost functions. The occupation measure can be interpreted as the probability of occupying state-action pairs in the long run, given that the PBN is initiated from state z_0_ and strategy *π* is used for the intervention. Consequently, the expected number of interventions in the long run can be constrained by the upper-bound ***C**_total_* if the cost-per-stage for each state-action pair is assigned as follows: *c(z,u)* = 0 if no intervention is applied and *c(z,u)* = 1 otherwise. This enables us to redefine the constrained optimization problem (4) as a linear program. An optimal constrained strategy of the intervention problem (4) can then be found based on a solution of this linear program.

If we assume that any gene in the mutated cell-cycle network could be used for therapeutic intervention, then it is natural to ask which gene would be the most effective lever point. To this end, we calculated constrained intervention strategies for each of the genes in the mutated cell-cycle network. The initial state is set to the undesirable state  with the highest long-run probability prior to intervention, and the upper bound of the frequency of applying interventions was varied. Table **3** lists the percentage change in the aggregated probability of undesirable states as a result of the intervention.

Among all the genes, *Rb* offers the best performance when intervention can be applied without any constraint ( *C_total_* = 1). Restricting the expected number of interventions to at most 10% and intervening again with *Rb*, the aggregated probability of undesirable states can be reduced to less than 12%. This information could be translated to restricting the dose density of a prescribed drug acting on *Rb* once its side effects are known. If we now slacken the limit on the expected number of applied interventions to, say, less than 30%, then we can reduce the chance of the cancerous states by 98%. The results of Table **3** demonstrate that the choice of the most effective control gene may vary depending on the restrictions imposed on the intervention. For instance, when the bound on the expected number of interventions is set to 30%, the constrained strategy based on *E2F* performs as well as the *Rb*-based constrained strategy.

## 6. INTERVENTIONS FOR ACCOMMODATING DIFFERENT BIOLOGICAL TIME SCALES

From a biological perspective, interactions among genes causing transcription, translation, and degradation occur over a wide range of time-scales. Earlier studies suggest that asynchronously updating the genes alters the global behavior of synchronous networks due to the change in their oriented graph, which models the dynamics of the system [16-18]. Synchronous abstraction is used under the implicit assumption that asynchronous updating will not unduly alter the properties of a system central to the application of interest [16]. Clearly, some properties will be altered. For instance, in Fig. (**2**), if all the genes are not simultaneously updated, then “010” may transition to some other state instead of transitioning to the attractor state “001”.

These observations motivate the examination of intervention in asynchronous models. Since relaxing the synchronous assumption alters the long-run behavior of  a regulatory model, alternative approaches are needed  to influence its dynamics. In [16], two new rule-based asynchronous models and methods to derive effective intervention strategies for each one of them are proposed. The first model introduces asynchronism in probabilistic Boolean networks at the gene level. This method is akin to our understanding from an interaction between biological components.

The second model extends PBNs by considering asynchronism at the state level. This approach is resourceful from a translational perspective. While the physical evolution of the biological gene network occurs over continuous time, the PBN records only the state transitions and contains no information on the time between the individual transitions. The PBN model inherits this property from the classical Boolean model, which it generalizes. Hence, the problem can be explained in the framework of the Boolean model. Figs. (**8**) and (**9**) show two continuous-time realizations that are equivalent from the point of view of the model of Fig. (**1**). In both Figs. (**8**) and (**9**), the initial state is  “100”. We observe the evolution “100” → “010” → “001”, at which point there are no other changes because “001” is an attractor of the network. While equivalent from the perspective of the Boolean model, from the perspective of continuous time observation, the realizations of Figs. (**8**) and (**9**) are not the same. For instance, in the second realization, the sojourn time in state “010” is much longer than in the first realization. This may be of no concern if we are only interested in tracking the state transitions. On the other hand, suppose we are considering intervention and penalizing undesirable states. Then, if “010” is an undesirable state,  the penalty should be greater in the second realization;  that is, the penalty needs to consider the sojourn time in  a state. This problem has been addressed in the framework of asynchronous Markovian regulatory networks by considering the process to be defined over continuous time.

### 6.1. Deterministic-Asynchronous Context-Sensitive PBNs

The first proposed asynchronous model, called a *deterministic-asynchronous context-sensitive probabilistic Boolean network* (DA-PBN), is an extension of probabilistic Boolean networks in which the time scales of the biological updates at various genes can be different, each gene being updated based on an individual period, which may differ from one gene to another. Yet, the updating period of  each gene is fixed given the context of the network. The  term “probabilistic” emphasizes the random selection of  a context, while the term “deterministic” refers to the deterministic asynchronous protocol within each context of the regulatory network. As a stochastic Boolean network with asynchronous updates, a DA-PBN expands the benefits of synchronous PBNs by adding the ability to cope with temporal context as well as regulatory context.

When the context of a biological system is known, asynchronism in regulatory networks is deterministic: that is, the updating period of each gene is fixed given the condition of the cell. However, *deterministic-asynchronous Boolean networks* pose practical challenges. Even if we can measure the level of each gene in isolation while the other genes remain constant, owing to the effects of measurement noise and the existence of latent variables, we cannot exactly specify the updating periods of genes. At best, we can estimate a set consisting of the most probable updating periods for each gene in the network, depending on the status of latent variables. A set of updating periods, whose members are the deterministic periods of each gene in the regulatory network, defines the updating protocol of a deterministic asynchronous Boolean network. This means that there is a finite collection of deterministic asynchronous Boolean networks that defines the dynamics of the system. The updating periods of genes depend on the temporal context of the biological system, which can be influenced by latent variables. The regulatory interactions among genes in a deterministic-asynchronous Boolean network are described similarly to a classical Boolean network.

In a DA-PBN, the behavior of latent variables influences both the regulation and the updating periods of genes. As with a synchronous PBN, uncertainty about the context  of a DA-PBN is captured through a probability distribution on the possible contexts, each being a deterministic-asynchronous Boolean network, which in turn describes the asynchronicity in the DA-PBN.

We resort to a synchronization method to intervene in a DA-PBN [16]. A price in terms of computational complexity has to be paid for synchronizing the model. This synchronization procedure translates the problem of intervention in a DA-PBN to infinite-horizon discrete-time sequential decision making. This mapping augments the state-space of a PBN, specified by the logical rules of the DA-PBN, with the necessary timing history of the DA-PBN. The augmented state-space has a considerably higher dimension. Now that the oriented graph of a DA-PBN is represented by a Markov chain with augmented state-space, all the previous intervention methods are applicable to this asynchronous model with slight modifications.

### 6.2. Semi-Markov Asynchronous Regulatory Networks

Assuming asynchronism at the gene level for Boolean networks poses practical and theoretical impediments that impede independent gene updating from serving as a basis for designing effective therapeutic interventions [16,32]. In particular, the delay and the updating order of a given gene are only observable in conjunction with the activity levels of other genes and proteins involved in the regulation process. Estimating the updating duration of each gene in isolation, independently of the values of other genes, is highly problematic, if not impossible. In practice, we can measure the aggregated values of all the genes (state) at each observation instant. The inter-transition interval between two states can then be modeled by a random variable.

Experimentally validated Boolean rules are considered in [26,32]. Under a synchronous assumption, the oriented graphs can accurately determine the phenotypic behavior of the underlying biological processes; however, these studies suggest that asynchronously updating the genes when utilizing the same Boolean rules generates very complex pathways which possess many incompatible and unrealistic phenotypes. It appears that asynchronously updating the individual genes changes the global behavior of the model by changing its oriented graphs.

Several studies suggest that rule-based regulatory models should maintain the topology of the oriented graph generated by experimentally validated predictor rules, as if the genes are coupled [26,32,33]. This is the type of information one can obtain from the biological literature and also in laboratory experiments. In other words, regulatory models should accurately manifest the logical relationships, i.e. the regulatory graph, governing the interactions of genes according to the gathered knowledge and translate them into the oriented graph specifying the dynamics of the disease. Moreover, they should enable the analysis of the temporal behavior of the pathological cellular functions.

Motivated by these observations, a *semi-Markov asynchronous regulatory network* (SM-ARN) is proposed in [16]. In an SM-ARN, asynchronism is at the level of aggregated gene expressions, i.e. states. In this model, the empirically measurable timing information of pathological cellular functions is incorporated into the model. This timing information determines the typical time delay between transitions from one state to another. The order of updating genes and their relative time delays depend on the levels of other regulatory components. Time-course data sets enable the estimation of inter-transition times between states, not the updating time of each gene in isolation.

An SM-ARN is specified with two sets of information. The first set determines the rule-based multivariate interactions between genes. Considering simultaneous updating, we can specify the oriented graph of the model based on this information. In other words, the first set of information specifies an *embedded-PBN*, which is generated from a given set of logical rules for updating each gene.  The generated oriented graph guarantees the predictability of the rule-based topology. The second set of information consists of the distributions of inter-transition intervals between any two states that are directly connected in the oriented graph. These can be empirically inferred from time-course data sets.

Designing optimal intervention strategies based on the SM-ARN model involves results from the theory of semi-Markov decision processes. Upon appropriately formulating the problem of intervention in the SM-ARN model, an effective intervention strategy can be devised. This strategy minimizes the time that the system spends in the undesirable states.

In [16], an SM-ARN is designed that models the dynamics of mutated mammalian cell-cycle regulation based on the logical rules described in Subsection 2.2, where it is assumed that the distribution of the inter-transition interval follows an exponential distribution. Therefore, we need the rate of the transition from state z_1_ to state z_2_ to specify their inter-transition interval distribution. Gene-expression data are used to determine the probability of the transition from state z_1_ to state z_2_ in the embedded-PBN. The rate of the transition from state z_1_ to state z_2_ can be defined from this probability [16]. The logical rules corresponding to the mutated scenario are used to construct the embedded-PBN of the cell-cycle's SM-ARN. The defined embedded-PBN maintains the topology of the oriented graph generated by the rules in Table **2**.

To avoid states with simultaneously down-regulated *CycD* and *Rb*, a semi-Markov decision process is used to spell out intervention strategies for the SM-ARN of the mutated cell-cycle. The rate of penalizing the states with down-regulated *Rb* and *CycD* is set to be higher than those for the states in which these two genes are not simultaneously down-regulated.

Fig. (**10**) depicts the fraction of time that the SM-ARN spends in each state when there is no intervention. Per this figure, the aggregated fraction of time that the mutated cell-cycle model spends in the states with simultaneously down-regulated *CycD* and *Rb* is 49%. The fraction of time that the SM-ARN of mutated mammalian cell cycle spends in states after *Rb*-based intervention is shown in Fig. (**11**). It is clear that after intervention using *Rb* as the control gene, the fraction of time that the model spends in the undesirable states is significantly reduced. Directly using *Rb* as the control gene, the fraction of time that the model spends  in the undesirable states is reduced to less than 2%. If  direct control based on *Rb* is not feasible, then one can use *E2F* as the control gene. In this case the system spends slightly more time in the undesirable states, but even this value is still less than 4.5%. Practically, the difference between the performances of these two control genes is insignificant. Figs. (**10**) and (**11**) lead us to the conclusion that the intervention method effectively alters the dynamics of the mutated cell-cycle SM-ARN.

## 7. CONCLUDING REMARKS

This paper has reviewed recent developments in the control of gene regulatory networks that aim to overcome engineering issues related to complexity, inference, and robustness, and also aim to develop intervention strategies commensurate with practical medical constraints. These developments have only begun to deal with these issues and much remains to be accomplished relative to these two aims. This paper provides a non-mathematical, biologically oriented treatment in the hope that it will generate further investigations on the part of biologists, engineers, mathematicians, and computer scientists interested in translational genomics.

## Figures and Tables

**Fig. (1):**
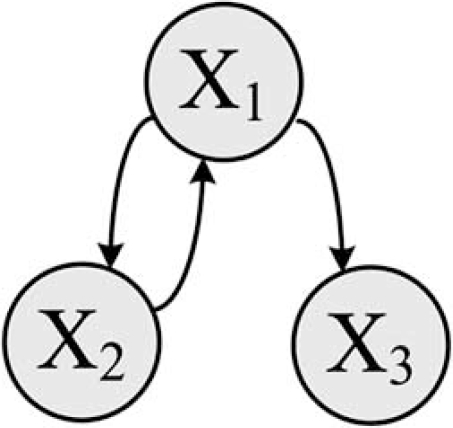
Presentation of a regulatory graph for an arbitrary 3-gene Boolean network.

**Fig. (2):**
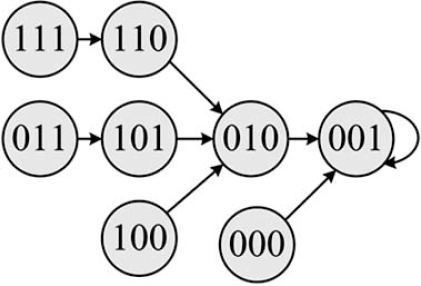
Presentation of the oriented graph corresponding to the 3-gene regulatory graph in Fig. (**1**).

**Fig. (3):**
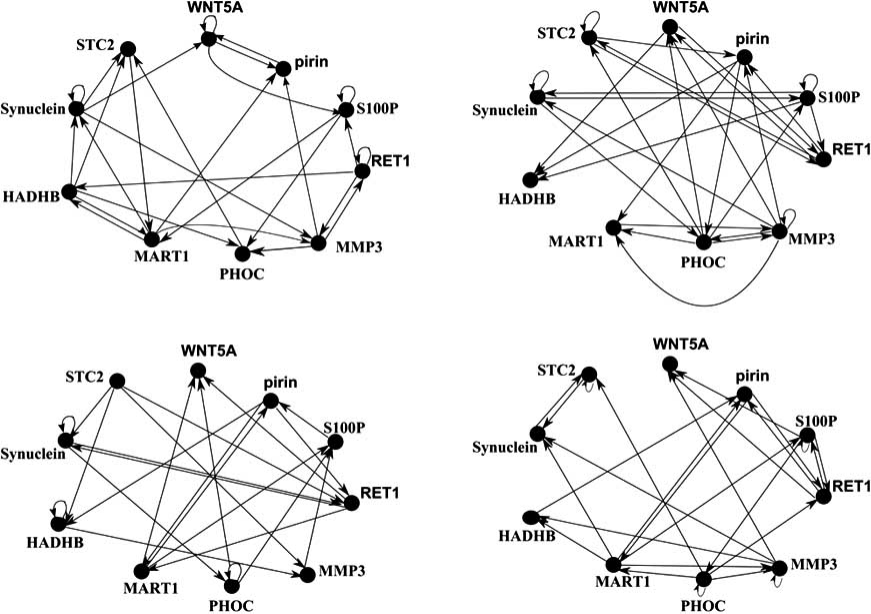
The regulatory graphs of the four constituent Boolean networks used to construct PBN for the metastatic melanoma data.

**Fig. (4):**
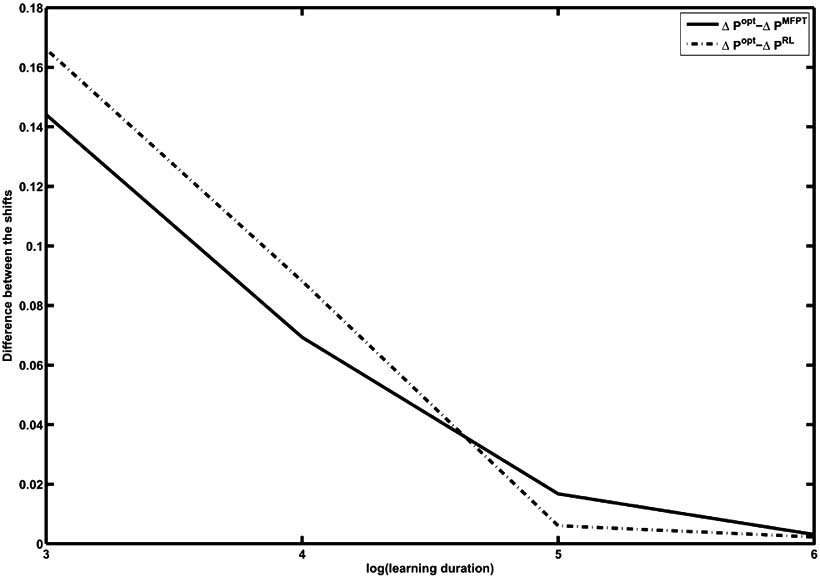
Comparison between the loss of performance in MFPT and RL methods with respect to classical intervention as a function of the log of the learning duration.

**Fig. (5):**
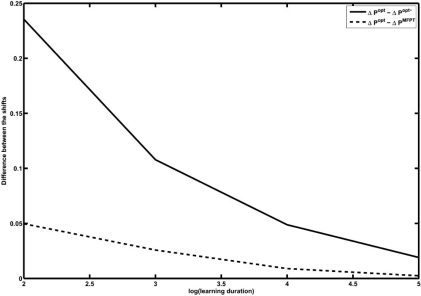
Average of Δ*P*^opt^-Δ*P*^opt*^ (solid) and Δ*P*^opt^ - Δ*P*^MFPT^ (dash) over 1000 trials as a function of the logarithm of learning duration.

**Fig. (6):**
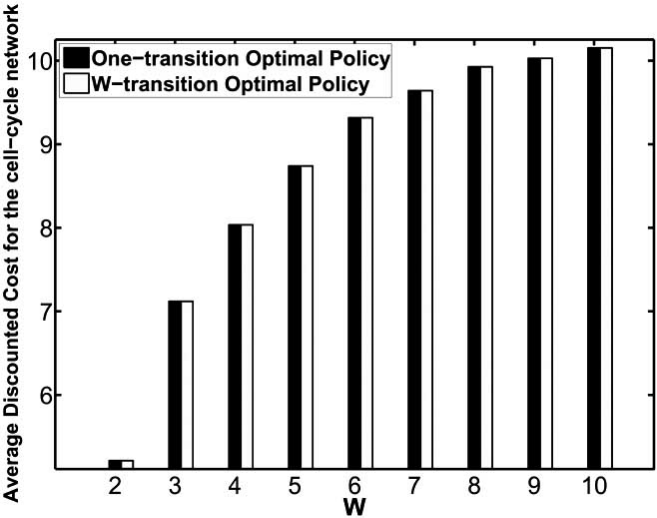
Comparison of the cyclic and classical strategies for treatment windows from 1 to 10 using the mutated mammalian cellcycle network: average total cost.

**Fig. (7):**
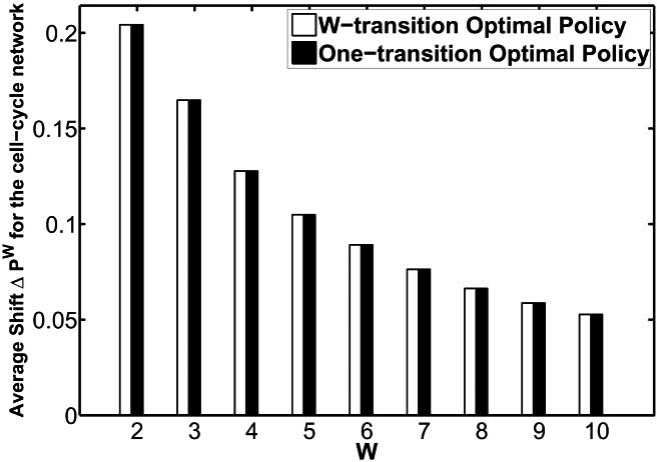
Comparison of the cyclic and classical strategies for treatment windows from 1 to 10 using the mutated mammalian cellcycle network: normalized change in the aggregated probability of undesirable states before and after the intervention.

**Fig. (8):**
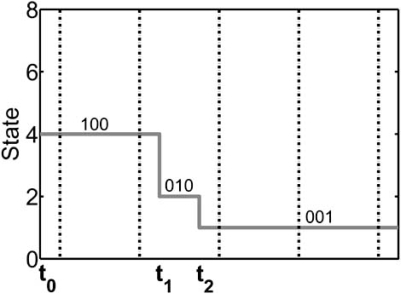
A realization of trajectories for the oriented graph in Fig. (2).

**Fig. (9):**
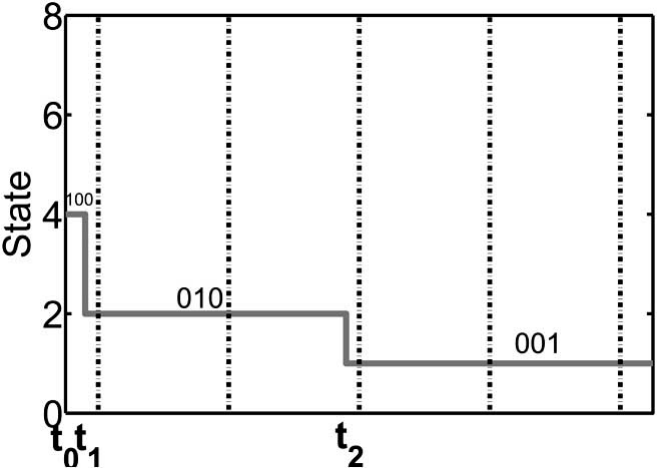
A realization of trajectories for the oriented graph in Fig. (2).

**Fig. (10):**
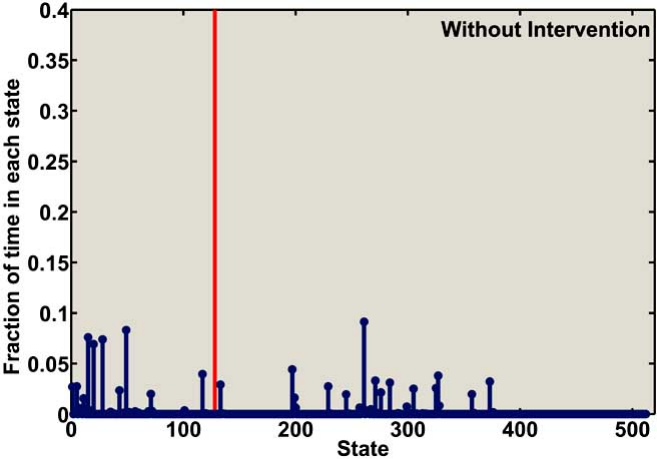
The fraction of time that the SM-ARN of mammalian cell cycle spends in each state prior to intervention. The vertical line separates the undesirable states from the desirable states.

**Fig. (11):**
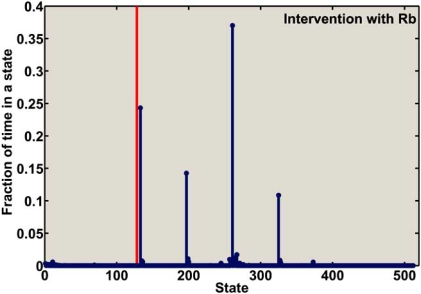
The fraction of time that the SM-ARN of mammalian cell cycle spends in states after intervention using *Rb* as the control gene. The vertical line separates the undesirable states from the desirable states.

**Table 1.** Wild-type Boolean Functions of Mammalian Cell Cycle

Product	Predictors
*CycD*	Input
*Rb*	$\left(\bar{\mathrm{CycD}}\wedge \bar{\mathrm{CycE}}\wedge \bar{\mathrm{CycA}}\wedge \bar{\mathrm{CycB}}\right)\vee \left(\mathrm{p27}\wedge \bar{\mathrm{CycD}}\wedge \bar{\mathrm{CycB}}\right)$
*E*2*F*	$\left(\bar{\mathrm{Rb}}\wedge \bar{\mathrm{CycA}}\wedge \bar{\mathrm{CycB}}\right)\vee \left(\mathrm{p27}\wedge \bar{\mathrm{Rb}}\wedge \bar{\mathrm{CycB}}\right)$
*CycE*	$\left(\mathrm{E2F}\wedge \bar{\mathrm{Rb}}\right)$
*CycA*	$\left(\mathrm{E2F}\wedge \bar{\mathrm{Rb}}\wedge \bar{\mathrm{Cdc20}}\wedge \left(\bar{\mathrm{Cdh1}\wedge \mathrm{UbcH10}}\right)\right)\vee \left(\mathrm{CycA}\wedge \bar{\mathrm{Rb}}\wedge \bar{\mathrm{Cdc20}}\wedge \left(\bar{\mathrm{Cdh1}\wedge \mathrm{UbcH10}}\right)\right)$
*p*27	$\left(\bar{\mathrm{CycD}}\wedge \bar{\mathrm{CycE}}\wedge \bar{\mathrm{CycA}}\wedge \bar{\mathrm{CycB}}\right)\vee \left(\mathrm{p27}\wedge \left(\bar{\mathrm{CycE}\wedge \mathrm{CycA}}\right)\wedge \bar{\mathrm{CycB}}\wedge \bar{\mathrm{CycD}}\right)$
*Cdc*20	*CycB*
*Cdh*1	$\left(\bar{\mathrm{CycA}}\wedge \bar{\mathrm{CycB}}\right)\vee \left(\mathrm{Cdc20}\right)$
*UbcH*10	$\left(\bar{\mathrm{Cdh1}}\right)\vee \left(\mathrm{Cdh1}\wedge \mathrm{UbcH10}\wedge \left(\mathrm{Cdc20}\vee \mathrm{CycA}\vee \mathrm{CycB}\right)\right)$
*CycB*	$\left(\bar{\mathrm{Cdc20}}\wedge \bar{\mathrm{Cdh1}}\right)$

**Table 2.** Mutated Boolean Functions of Mammalian Cell Cycle

Product	Predictors
*CycD*	Input
*Rb*	$\left(\bar{\mathrm{CycD}}\wedge \bar{\mathrm{CycE}}\wedge \bar{\mathrm{CycA}}\wedge \bar{\mathrm{CycB}}\right)$
*E*2*F*	$\left(\bar{\mathrm{Rb}}\wedge \bar{\mathrm{CycA}}\wedge \bar{\mathrm{CycB}}\right)$
*CycE*	$\left(\mathrm{E2F}\wedge \bar{\mathrm{Rb}}\right)$
*CycA*	$\left(\mathrm{E2F}\wedge \bar{\mathrm{Rb}}\wedge \bar{\mathrm{Cdc20}}\wedge \left(\bar{\mathrm{Cdh1}\wedge \mathrm{UbcH10}}\right)\right)\vee \left(\mathrm{CycA}\wedge \bar{\mathrm{Rb}}\wedge \bar{\mathrm{Cdc20}}\wedge \left(\bar{\mathrm{Cdh1}\wedge \mathrm{UbcH10}}\right)\right)$
*Cdc*20	*CycB*
*Cdh*1	$\left(\bar{\mathrm{CycA}}\wedge \bar{\mathrm{CycB}}\right)\vee \left(\mathrm{Cdc20}\right)$
*UbcH*10	$\left(\bar{\mathrm{Cdh1}}\right)\vee \left(\mathrm{Cdh1}\wedge \mathrm{UbcH10}\wedge \left(\mathrm{Cdc20}\vee \mathrm{CycA}\vee \mathrm{CycB}\right)\right)$
*CycB*	$\left(\bar{\mathrm{Cdc20}}\wedge \bar{\mathrm{Cdh1}}\right)$

**Table 3.** The Percentage Change in the Aggregated Probability of States with Down-Regulated *CycD* and *Rb* Based on Various Control Genes and Constraint Bounds.

Control Gene	*C_total_*			
	0.1	0.3	0.5	1.0
*Rb*	61.96	98.33	98.33	98.34
*E* 2 *F*	57.43	98.00	98.00	98.02
*CycE*	28.37	28.41	28.44	28.51
*CycA*	16.56	16.60	16.62	16.69
*Cdc* 20	39.15	41.47	41.48	41.61
*Cdh* 1	27.55	41.51	41.56	41.65
*UbcH*10	6.49	6.52	6.57	6.69
*CycB*	39.33	41.86	41.91	41.99
